# Protozoa population and carbohydrate fermentation in sheep fed diet with different plant additives

**DOI:** 10.5713/ajas.20.0477

**Published:** 2020-10-14

**Authors:** Małgorzata P. Majewska, Renata Miltko, Grzegorz Bełżecki, Aneta Kędzierska, Barbara Kowalik

**Affiliations:** 1 Department of Animal Nutrition, The Kielanowski Institute of Animal Physiology and Nutrition, Polish Academy of Sciences, Jabłonna 05-110, Poland

**Keywords:** Lingonberry Leaves, Oak Bark, Microorganisms, Short-chain Fatty Acids, Methane, Sheep

## Abstract

**Objective:**

The aim of the study was to compare the effect of two plant additives, rich in polyphenolic compounds, supplemented to sheep diets on microorganisms and carbohydrate fermentation in rumen.

**Methods:**

In the experiment, 6 ewes of the Polish Mountain breed were fitted with ruminal cannulas. Sheep were divided into three feeding groups. The study was performed in a cross-over design of two animals in each group, with three experimental periods (n = 6 per each group). The animals were fed a control diet (CON) or additionally received 3 g of dry and milled lingonberry leaves (VVI) or oak bark (QUE). Additionally, plant material was analyzed for tannins concentration.

**Results:**

Regardless of sampling time, QUE diet increased the number of total protozoa, as well as *Entodinium* spp., *Diplodinium* spp. and *Isotrichidae* family, while decreased bacterial mass. In turn, a reduced number of *Diplodinium* spp. and increased *Ophryoscolex* spp. population were noted in VVI fed sheep. During whole sampling time (0, 2, 4, and 8 h), the number of protozoa in ruminal fluid of QUE sheep was gradually reduced as opposed to animals receiving CON and VVI diet, where rapid shifts in the protozoa number were observed. Moreover, supplementing sheep with QUE diet increased molar proportions of butyrate and isoacids in ruminal fluid. Unfortunately, none of the tested additives affected gas production.

**Conclusion:**

The addition of VVI or QUE in a small dose to sheep diets differently affected rumen microorganisms and fermentation parameters, probably because of various contribution of catechins in tested plant materials. However, it is stated that QUE diet seems to create more favorable conditions for growth and development of ciliates. Nonetheless, the results of the present study showed that VVI and QUE additives could serve as potential natural modulators of microorganism populations and, consequently, carbohydrate digestion in ruminants.

## INTRODUCTION

Unlike monogastric animals, the presence of the rumen, which acts as a fermentation chamber, enables ruminants to utilize roughage as a source of energy. Additionally, ruminants do not produce enzymes responsible for degrading fibrous plant material. Thus, digestion in the rumen occurs only due to the symbiotic relationship of bacteria, protozoa, and fungi with the host organism [1]. Interestingly, the composition of rumen microorganisms may be affected by several factors, including diet composition, age, health of the host animal, as well as geographical location and feed regimen [2].

Recently, scientists have been seeking natural agents in ruminants’ nutrition, which may directly or indirectly modify the ruminal microorganism population as well as carbohydrate fermentation and methanogenesis. The example of such factors can be herbal additives, including lingonberry leaves and oak bark, with properties applicable in medicine. Lingonberry (*Vaccinium vitis-idaea* L.) is an eminent fruit crop with health promoting properties. Interestingly, leaves and berries of lingonberry contain a lot of polyphenolic compounds, which exert high antioxidant activities [3]. Oak (*Quercus* sp.) is a popular wood in the cooperage industry and in the barrels and stoppers production. Until recently, oak bark was treated as a waste of wood transformation with scarce application and relatively low price [4]. But similar to aforesaid lingonberry, it is a valuable source of polyphenols. It became popular in traditional medicine for treating different illnesses and wounds because of its antioxidant, antibacterial and anti-inflammatory activities [3].

The common feature of both plants is the presence of secondary metabolites, for instance, tannins. Generally, tannins are characterized as water-soluble polyphenols of various molecular mass, commonly found in nature [5]. Due to their different molecular weights, they may exhibit different reactivity and biological activity. On this basis, such compounds are divided into two main groups: hydrolysable and condensed tannins [5]. Kylli et al [6] reported that the main phenolic compounds found in lingonberries are proanthocyjanidins (condensed tannins), which consist of catechin, epicatechin, gallocatechin and epigallocatechin units. By contrast, in oak bark, hydrolysable tannins (including gallo- and ellagitannins) constitute the overwhelming number of polyphenolic compounds, while complex tannins as well as proanthocyjanidins (oligomers and polymers) are in the minority [4].

Several studies have documented that the usage of plants containing tannins in ruminants’ diet may affect microorganisms number, digestive enzymes, the degradability of diet components and, consequently, nutrient availability in other parts of the digestive tract [7–9]. Nonetheless, it is worth noting that tannins are generally classified as anti-nutritional compounds, which may considerably limit their use in animal nutrition. For that reason, their contribution in the diets should be monitored with the greatest caution.

Interestingly, a considerable part of the researches on bio active compounds of natural additives were *in vitro* studies, while studies performed on ruminants are often limited. Unfortunately, information about the effect of lingonberry leaves and oak bark addition, on rumen microorganisms and fermentation parameters in ruminants are still scarce.

It was hypothesized that supplementing sheep diets with lingonberry leaves and oak bark, may affect microorganism populations as well as end products of ruminal carbohydrate fermentation.

Hence, the aim of the present study was to determine the effect of two natural additives, differing in polyphenolic compounds contents (tannins), to sheep diets on rumen microorganisms and carbohydrate fermentation parameters. To identify the whole appearing changes in time due, protozoa number, pH of rumen fluid, the molar proportion of short chain fatty acid (SCFA) as well as estimated gas production were measured before feeding and at 2, 4, and 8 hours after feeding, whereas samples for bacterial biomass estimation were pooled.

## MATERIALS AND METHODS

All procedures performed in the present study were accepted by the Local Animal Care and Ethics Committee for Animal Experiment in Warsaw (Poland); permission no. 51/2009.

### Animals and diets

The experiment was carried out on 6 ewes of the Polish Mountain breed with an average body weight of 33±1.1 kg, fitted with rumen cannulas. The rumen fistula was placed in the dorsal part of the rumen sac on the left side of the body. Sheep were housed in the individual pens, padded with straw, with *ad libitum* access to water and salt licks.

The experiment was performed in a cross-over design of two animals in each group with three periods (n = 6 per group; Table 1). Each period of the experiment lasted 37 days, including 14 days of gradual transition to the diet, 21 days of adaptation to the specific diet and 2 days of sampling. The control diet (CON) consisted of meadow hay, barley meal, soybean meal and vitamin-mineral premix (Table 2). Sheep in experimental groups additionally received 3 g of dry and milled lingonberry leaves (*Vaccinium vitis-idaea* L., VVI) or oak bark (*Quercus* sp., QUE), which were thoroughly mixed with the control diet before administration. The animals were fed twice a day at 7.00 am and 15.00 pm. Plant material was analyzed for tannins concentrations. The amount of tannins in the preparations was 10.3 and 4.0 g of catechins equivalent per kg of dry matter (DM) of the additive, for VVI and QUE respectively [10]. The contribution of components in the DM of feed and their chemical composition is presented in Table 2. The energy value of the food was calculated based on feeding standards for ruminants IZ PIB-INRA [11]. The DM uptake of control diet was 919 g per day.

### Rumen fluid sampling

The collection of rumen fluid was performed for two consecutive days. Rumen fluid samples (about 270 mL) were taken before feeding and at 2, 4, and 8 hours after feeding. The rumen fluid was filtrated through a complex of 4 layers of surgical gauze and its pH, protozoan count and SCFA concentration were determined.

### Analytical methods

The chemical composition of feed, including DM (934.01), crude protein (954.01), crude fiber (978.10), acid detergent fibre and acid detergent lignin (973.18), neutral detergent fibre (2002.04), starch (920.4) and crude ash (930.05) contents, was determined according to the AOAC method [12].

*Protozoa number and bacterial mass*: For the determination of protozoan number, 5 mL of rumen fluid were fixed with 10 mL of 4% formaldehyde solution. Such samples were stored in tightly closed containers at 4°C until analysis. The number of protozoa was identified and classified according to Dogiel [13] and determined using a light microscope according to the method of Miltko et al [14].

Rumen bacterial biomass was determined according to the method of Michałowski et al [15]. For this measurement unfiltered rumen fluid samples were used, so as not to remove bacteria attached to the feed particles accidentally. Briefly, the rumen fluid was centrifuged in the three-step centrifugations. The first centrifugation lasted 10 minutes at 600×g, which allowed removal of protozoa and feed residues (if present). The collected supernatant was subjected to centrifugation for another 30 minutes at 30,000×g. The sediment was dissolved in 0.9% saline solution and centrifuged once again at the same parameters as previous centrifugation to obtain purified bacteria material. After last centrifugation, bacterial pellet was dissolved in 5 mL of 0.9% saline solution to accurately transfer it from the tube and weighed. Three-step centrifugations for all sampling times (before feeding and at 2, 4, 8 h after feeding) were performed. Due to the small amounts of bacterial mass obtained from the ruminal fluid, the samples from different time points were pooled and expressed as g/8 h, taking into account the known volumes of rumen fluid and salt solution used during centrifugation, as follows:

$$\text{Bacterial mass }(\text{g}/8\;\text{h})=\frac{\text{sum of bacterial mass }(0,2,4,8\;\text{h})-20\;\text{mL of saline solution}}{\text{sum of rumen fluid }(0,2,4,8\;\text{h})}\times 100$$

According to the above equation, the sum of bacterial mass, volumes of rumen fluid and saline solution at different time points were used. Finally, the bacterial pellets of each animal from CON, VVI, and QUE groups were dissolved in 0.9% saline solution and stored at −24°C.

*pH and short-chain fatty acids*: The acidity of rumen fluid was immediately determined with a F72 Beckman pH-meter during sampling time.

For the determination of SCFA, 5 mL of rumen fluid were treated with 0.5 mL of 85% of formic acid, then centrifuged at 11,000×g for 25 min and finally stored in sealed vials at 4°C until analysis. One ml of 4-methylvaleric acid in 100 mL of water solution was added to each sample and served as an internal standard during analysis. The concentrations of SCFA in the rumen fluid samples were determined using a gas chromatograph (Shimadzu GC-2010, Tokyo, Japan) equipped with a 30 m capillary column (0.25 mm i.d. ×0.25 μm film thickness) and a flame ionisation detector, with helium as a carrier gas, according to the method of Miltko et al [16].

*Gas production*: Based on the concentrations of specific SCFA, the quantification of methane and carbon dioxide content in the rumen fluid of sheep according to the molar proportions of SCFA was provided (a theoretical fermentation balance) [17]:

$$57.5\;({\text{C}}_{6}{\text{H}}_{12}{\text{O}}_{6})\to 65\;\text{acetate }({\text{C}}_{2})+20\;\text{propionate }({\text{C}}_{3})+15\;\text{butyrate }({\text{C}}_{4})+60\;{\text{CO}}_{2}+35\;{\text{CH}}_{4}+25\;{\text{H}}_{2}\text{O}.$$

According to above reaction (i.e. the ruminal microbial fermentation), for a molar distribution of 65 acetate: 20 propionate: 15 butyrate (65C_2_:20C_3_:15C_4_), 60 moles of CO_2_ and 35 moles of CH_4_ are formed. Thus, based on this equation, the following amounts of the individual acids are generated from 1 mole of glucose:

$$1.13\;{\text{C}}_{2}+0.3478\;{\text{C}}_{3}+0.2608\;{\text{C}}_{4}+1.043479\;{\text{CO}}_{2}+0.608696\;{\text{CH}}_{4}.$$

Therefore, the theoretical molar proportions as well as theoretical concentrations of microbiota fermentation products are as follows:

$$1\text{M }{\text{C}}_{2}+0.307787\text{M }{\text{C}}_{3}+0.2308\text{M }{\text{C}}_{4}+0.9234327\text{M }{\text{CO}}_{2}+0.5386690265\text{M }{\text{CH}}_{4}.$$

Based on the experimental concentrations of C_2_, C_3_, and C_4_ obtained in the present study (mM/100 mL), the critical concentrations of C_2_, C_3_, or C_4_ should be found. In fact, in the analyzed ovine ruminal fluid, the concentrations of two SCFA (i.e. C_2_, C_3_, or C_4_) were generally higher than the theoretical concentrations of C_2_, C_3_, or C_4_ [17]. Importantly, the critical concentrations of these acids determined the amounts of CH_4_ and CO_2_ generated (mM/100 mL), according to the fermentation reaction. The critical concentrations of aforesaid acids have been designated based on the obtained experimental data by dividing the concentrations of these acids with each other in the Microsoft Excel program.

### Statistical analysis

The results of the current study were presented as means and pooled standard error of mean (SEM). To assess the normality of the distribution of the obtained data, the Shapiro-Wilk test was used. Additionally, the homogeneity of variances was also checked by Levene’s test. Data with abnormal distribution were transformed into natural logarithms. The results were subjected to two-way analysis of variance (ANOVA) (with two factors: diet and sampling time; including protozoa number, pH, SCFA concentration and gas production), or one-way ANOVA (one factor: diet; including bacterial biomass). The significance of both: i) interactions between diet and sampling time (two-way ANOVA) and/or ii) differences between dietary treatments (one-way ANOVA) was determined by a Tukey test. The differences were considered significant at p≤0.05. However, differences at p>0.05 were defined as trends. The statistical analysis was performed using the Statistica program (StatSoft, Polska, 2011).

## RESULTS

### Rumen microorganisms

A significant interaction between diet and sampling time were noted for total protozoa number and their species (p≤0.001; Table 3). However, the effect of plant additives on protozoa population was distinct.

Supplementing sheep with QUE addition significantly in creased total number of protozoa and *Entodinium* spp. at 2 and 4 hours after feeding, and, simultaneously reduced their number at 8 hours after feeding, compared to the CON and VVI groups (p≤0.001). Furthermore, feeding sheep with QUE diet also significantly increased the *Diplodinium* spp. population before feeding (vs VVI group) as well as at 2 (vs CON group) and 4 hours after feeding (vs VVI diet) and reduced their number at 8 hours after feeding, compared to the CON and VVI groups. A greater number of protozoa of the *Dasytricha* genus was found in QUE sheep at 2 and 4 hours after feeding in comparison to the CON and VVI groups, while reduction of their population was showed at 8 hours after feeding, compared to the CON group (p≤0.001).

The addition of VVI to sheep diets significantly reduced the number of *Diplodinium* spp. population before and at 4 hours after feeding in comparison to the CON and QUE groups, while increased their number at 8 hours after feeding in comparison to QUE sheep (p≤0.001). Furthermore, there were significantly increased numbers of *Ophryoscolex* spp. protozoa in VVI sheep before feeding and at 2 hours after feeding, compared to the CON group, as well as at 8 hours in comparison to the QUE group.

Both plant additives significantly increased the *Isotricha* spp. number before feeding, compared to the CON sheep (p≤0.001), while then some changes in time due were noted. Supplementing sheep with QUE diet increased *Isotricha* spp. population at 2 hours and reduced their number at 8 hours after feeding in comparison to the CON and VVI groups.

Worth noting was fact that all changes in protozoa number after feeding time in QUE sheep were not as spectacular as observed regarding to the CON and VVI groups. It means that during whole sampling time (0, 2, 4, and 8 h), the number of protozoa in ruminal fluid of sheep receiving QUE diet was gradually reduced (p≤0.001). On the contrary, drastic reduction of protozoa number was reported at 2 and 4 h after feeding in CON and VVI animals and then slightly increased at 8 h after feeding (p≤0.001).

Feeding sheep with QUE diet significantly reduced bacterial mass in rumen fluid in comparison to VVI diet (p = 0.003; Table 3).

### Indicators of rumen fermentation and gas production

Any significant interaction between diet and sampling time of ruminal fluid pH in present study was observed (p = 0.194; Table 4).

Likewise, the addition of both natural additives to sheep diets did not significantly affect the concentration of total SCFA (p = 0.895), as well as molar proportions of acetate (p = 0.337), propionate (p = 0.234) and valerate (p = 0.182) in rumen fluid before and after feeding the animals.

However, significant interaction between diet and time for molar proportion of butyrate was noted (p = 0.011). Briefly, higher values of butyrate were documented for QUE sheep before feeding (vs VVI group), at 2 and 4 h after feeding (vs CON and VVI sheep) as well as at 8 h when compared to VVI group (p≤0.001).

Similar dependencies were shown for molar proportions of isoacids, including isobutyric and isovaleric acids (p≤ 0.001). Higher values of such acids were noted in sheep receiving QUE diet at 2 and 4 h after feeding in comparison to CON and VVI groups (p≤0.001).

Interestingly, some different changes in molar proportions of butyrate and isoacids in time due were observed. Generally, when adding both plant sources to sheep diets, the molar proportion of butyrate after feeding (p = 0.066) was rather constant. On the contrary, the amount of isoacids in rumen fluid decreased after feeding time, regardless of dietary treatments (p≤0.001).

The addition of VVI and QUE to sheep diet did not significantly affect methane and carbon dioxide concentrations in rumen fluid (p = 0.158; Table 5).

## DISCUSSION

The information about quantity, form (part of the plants, pure extract) as well as the presence of phenolic compounds is of utmost importance, when interpreting the results of studies using phyto-sources in animal nutrition. It is often because biologically active substances found in plant materials may differ in their properties and reactivity.

### Rumen microorganisms

In ruminants, microorganisms habituating rumen play an important role and are involved in nutrient digestion [1]. Bioactive compounds present in a fodder may directly affect the growth and activity of both rumen microflora and microfauna, as well as nutrient availability, which determine their population [8]. All changes in the microorganism populations influence digestion processes in rumen and finally the amount and type of nutrients reaching further parts of the digestive tract of ruminants. According to the systematics of Dogiel [13], the protozoa found in the rumen belong to the two main families: *Ophryoscolecidae* and *Isotrichidae*. The first family is represented by three genera of protozoa, including *Entodinium* spp., *Diplodinium* spp. and *Ophryoscolex* spp., and the second by two genera, namely *Isotricha* spp. and *Dasytricha* ssp. In the present study, both VVI and QUE diets significantly affected protozoa count in the sheep rumen fluid. The largest group of protozoa were *Entodinium* genus in all dietary treatments. Additionally, rapid shifts in protozoa number was recorded for CON and VVI diets. On the contrary, the supplementation of QUE additive to sheep diet seems to have more stabilizing impact on the protozoa population. Such various effects of plant additives may probably be the result of different polyphenolic compounds content present in animal diets. It was shown that VVI diet contained up to twice higher concentration of catechins (condensed tannins) than the QUE diet. Smeriglio et al [18] reported that, the predominant components of oak bark (*Quercus robur* L.) are hydrolysable tannins, while the contribution of proanthocyanidins (condensed tannins) is rather low, as shown in the present study. Thus, different types of tannins may be probably a significant factor affecting protozoa population. However, we cannot exclude the effect of other biologically active compounds found in such additives on protozoa number. For example, comparing the concentration of various compounds present in lingonberry leaves, up to 12% content is reported for tannins, while approximately 5% to 7% constitutes for arbutin and methylarbutin (phenolic glycosides) [3]. In sheep supplemented with VVI diet an increased number of *Ophryoscolex* spp. was noted. Such protozoa are mainly involved in the digestion of structural carbohydrates, including cellulose, hemicellulose, and pectin, while simple sugars are used only in small amounts [19]. Conversely, in sheep receiving QUE diet, an increased total number of protozoa, as well as *Entodinium* spp., *Isotricha* spp. and *Dasytricha* ssp., which utilise easily digestible carbohydrates, including starch [19], was observed. Interestingly, an increased number of protozoa may be considered to be an indicator of ruminants’ health [20]. In particular, the presence of protozoa has a buffering effect on rumen conditions by engulfing starch granules and slowing down its fermentation, which protects the animal from rapid fluctuations of ruminal pH and may prevent acidosis [19]. This is of great importance for the animal because microorganisms habituating rumen are sensitive to the pH changes. Rapid increase or decrease of pH value can lead to growth and development of specific groups of microorganisms at the expense of others, and thus may significantly alter or even disrupt the digestion of nutrients as well as fermentation pattern. Importantly, the ability of protozoa to detoxify toxins taken up by the host organism from the feed is also known [19].

Interestingly, only QUE diet decreased bacterial mass in the rumen fluid. Looking at the rumen microorganisms and their interdependencies as a whole, the smaller amount of bacteria may be derived from a higher number of protozoa, which engulf bacterial cells and, thus, can modify the size of their population [19]. Bacterial cells are rich source of nitrogenous compounds, which are necessary for protozoan growth and development. Interestingly, comparative studies on faunated and defaunated (ciliate-free) sheep receiving the same diet, showed that predatory activity of protozoa reduced bacterial population by up to 50% to 90% primarily in faunated animals [19]. At this moment, it is difficult to propose other more accurate mechanisms of tannins action on an increased number of protozoa. The results of the studies exploring effect of tannins on bacterial population seem to be more explicit than for protozoa. It was shown that bacteria have developed some adaptive mechanisms to tannins, for instance the presence of tannase, enzyme responsible for the hydrolysis of the ester binding of tannic acid between gallic acid and glucose [21]. As shown in the study of Patra et al [21], hydrolysable tannins can be degraded by microorganisms into gallic acid, pyrogallol and phloroglucinol, and then to SCFA. Therefore, it could be possible that some rumen microorganisms have adapted to the presence of hydrolysable tannins in the QUE additive. We also think that QUE diet may create more favorable conditions for the development of some protozoa in the rumen. In fact, it could be possible that bacteria constituted the first line of defense and resist the toxic effect of tannins, indirectly affecting protozoa number. Importantly, it is not excluded that protozoa may also have some mechanisms of adapting to tannins, similar to bacteria. Our suspicions seem to remain only in the sphere of deliberation so far. Thus, this issue still requires more detailed studies both *in vivo* and *in vitro*. Unfortunately, in the present study, only bacterial mass was determined. Further subsequent microbiological studies using modern molecular techniques, like real-time polymerase chain reaction (real-time PCR) or polymerase chain reaction-denaturing gradient gel electrophoresis (PCR-DGGE), seem to be necessary to explore the effect of tested plant sources on different bacteria species and finally to know if the impact of tannins on bacteria is equal or rather species dependent.

Generally, in the literature, most studies explore the effect of plants containing primarily condensed tannins on rumen microorganisms and the great majority of them were conducted under *in vitro* conditions. Thus, the results of these researches are often controversial and seem to depend both on the different origin of such compounds and their destination. Thereby, it is of great importance to determine whether tannin-rich plants are: i) used as additives; ii) partially incorporated to the diet, replacing other components; or whether iii) they are offered to the animals as their sole feed. Most studies documented anti-protozoic [22–24] and anti-bacterial [22,25] actions of various tannin-rich plants addition. Furthermore, Aghamohamadi et al [26] reported decreased numbers of *Entodinium* spp. and *Ophryoscolex* spp. protozoa, as well as cellulolytic bacteria, after supplementing sheep diet with oak acorn (*Quercus perlica*). Interestingly, Carulla et al [27] noted that *Isotrichidae* protozoa seem to be more sensitive to the action of condensed tannins from *Acacia mearnsii* extract in comparison to *Entodinium* spp. This effect was not observed in the present study. Similarly, to our observations, Chiquette et al [28] showed increased protozoa number in rumen of sheep after *Lotus corniculatus* and *Hedysarum coronarium* (condensed tannins) supplementation. Furthermore, in sheep grazing sulla (*Hedysarum coronarium*) the protozoal number was also increased [29]. Interestingly, the addition of herbal preparation Ruchamax (consisting of 26 different plant extracts) to heifers’ diet, significantly increased total protozoa and *Entodinium* spp. numbers [20] as documented in the current study. On the other hand, there are several studies, which did not show any effect of tannin-rich plants addition on the protozoa population [28,29]. According to above mentioned studies, noteworthy is fact that origin of tannins, can be just as crucial as their types in affecting the number of rumen microfauna.

### Indicators of rumen fermentation

In the present study, the pH of rumen fluid was within normal range and none of the additions affected its value. The obtained results agree with Śliwiński et al [30], who did not show any differences in pH after feeding lambs with extract from *Castanea sativa* (hydrolysable tannins).

Rumen protozoa play an important role in carbohydrate metabolism, but depending on the species composition different fermentation patterns can be observed [19]. Generally, protozoa are believed to be one of the major producers of butyric acid in the rumen [19]. Michałowski [31] indicated that during ruminal carbohydrate fermentation, protozoa and bacteria produced total SCFA with similar intensity, but the main differences were observed in the concentrations of butyric and propionic acids. The formation of acetate was similar in both groups and accounted for over 50% of total SCFA [31]. Comparing molar concentrations of particular SCFA produced by different groups of microorganisms, Michałowski [31] stated that rumen microfauna were responsible for two-fold greater production of butyrate in the rumen of sheep than bacteria (41.3% vs 20.4% on average, respectively). Bacteria, in turn, produced higher concentrations of propionate to protozoa (22.1% vs 6.7% on average, respectively) [31]. Therefore, the composition of microorganisms in the rumen may significantly affect the carbohydrate fermentation pattern. Considering the above facts, it seems that higher molar proportion of butyrate in QUE sheep may primarily result from an increase in total protozoa number. Similar dependencies were also observed in the study of Terrill et al [29] in sheep grazing sulla. Additionally, it cannot be excluded that part of the butyrate in rumen fluid may originate from the microbial degradation of hydrolysable tannins [21]. In turn, elevated concentrations of branched-chain fatty acids (BCFA) in QUE diet may indicate impact of tannins on the rate of dietary protein degradation as well as deamination process. Indeed, rumen bacteria require BCFA to synthesize the carbon skeleton of branched-chain amino acids and long-chain fatty acids [32].

In the literature, the various effects of plant additives on carbohydrate fermentation, which certainly depend on plant materials, their dosage added to ruminants’ diet, as well as the presence of various biologically active compounds are documented. On the one hand, several *in vitro* and *in vivo* studies showed that the addition of tanniniferous plant materials did not significantly influence SCFA concentrations in rumen [26,33]. On the other hand, some researches have demonstrated some changes regarding total SCFA concentrations as well as their individual types. The study of Chanthakhoun et al [34] showed that feeding swamp buffalo with legume (*Phaseolus calcaratus*) hay significantly increased SCFA concentration, as well as acetic and propionic acids in rumen. Contrary to the present study, Cieślak et al [23,24] observed an increased concentration of propionic acid and lower acetic to propionic ratio, after using extract from lingonberry or oak bark, which may suggest negative impact of tannins on fiber digestion and cellulolytic microorganisms activity. In the current study, dry plant materials (not extracts) were used. Thus, despite the use of similar plant additives, the final amount of biologically active compounds was lower in a raw product than after extraction, which may partially explain observed differences [3]. By contrast, sheep receiving extract from *Acacia mearnsii* (condensed tannins) had higher concentrations of butyric and valeric acids in rumen [27]. Similar dependencies were also documented in studies on cows fed with plants containing condensed tannins [35], which were partially confirmed by the results obtained for QUE sheep. In study of Doce et al [36], an increased concentrations of butyric acid in rumen fluid of steers fed diet with different doses of oak leaves (*Quercus pyrenaica*, hydrolysable tannins) were also documented, which is in agreement with present study.

According to the literature, the production of methane in ruminants mainly depends on diet and composition of microorganisms habituating rumen. Protozoa are important producers of molecular hydrogen in rumen [37], which, in turn, is utilized by methanogens to methane production. Interestingly, methanogen communities can be associated with protozoa or may live in proximity to the protozoan cells [38]. Thus, every single disruption in the protozoa-methanogens relation, may affect the scale of methane production. Hegarty [39], summarizing the results of both *in vivo* and *in vitro* studies, showed significant reduction or even complete elimination of the protozoan population in the rumen by diet with high proportion of concentrate, which resulted in a diminution of methane production by approximately 13%. According to the authors, such changes arise because of reduced digestibility of DM, smaller population of methanogens (lower availability of hydrogen) and changes in the proportion of SCFA. Importantly, the inhibition of methanogenesis process by partial or complete elimination (defaunation) of the protozoa population (as hydrogen donors) in the rumen have been demonstrated by several authors [37,40–42]. Recently, studies focusing on the mechanism of methanogenesis reduction by dietary treatments, appear with increasing frequency. Cieślak et al [23,24] and Hess et al [43], reported lower methane production after supplementing animal feed with extracts originating from different plants (lingonberry, oak bark and *Acacia mearnsii* respectively). As reported by authors, the inhibition of methanogenesis was probably caused by the lower number of protozoa and simultaneously increased concentration of propionic acid (methane antagonist) [23,24] by limited ruminal fiber digestion [36].

Unfortunately, despite reported changes in the microorganism population in sheep fed diets with VVI and QUE, the amount of the additives used in the present study seems to be not effective in lowering methane and carbon dioxide concentrations in the rumen. Similar observations were reported in the Krueger et al [35] study, where the addition of mimosa (condensed tannins) or chestnut (hydrolysable tannins) extracts to cow feed rations did not have any significant effect on rumen methane production. It is worth noting that in the present study gas production was estimated according to the stoichiometric calculations of SCFA production, due to the mutual relationships between product and substrate of fermentation reaction [17]. It is a simple way to measure methane and carbon dioxide where it is not physically possible to detect the gases released from the animal, for example in respiration chambers. The accuracy of methane prediction from SCFA concentrations was raised by Wolin [17], who documented that values obtained from the theoretical predictions agree with experimental values. Similarly, the study of Whitelaw et al [44] on steers showed that stoichiometric calculations of methane production from the proportions of SCFA were of the same order of magnitude as results obtained in the respiration chambers. Interestingly, the values obtained from theoretical predictions overestimated methane production by 1.08 on average.

## CONCLUSION

In the present study, small doses of VVI or QUE addition to sheep diets differently affected rumen microorganisms and fermentation parameters. Additionally, QUE diet seems to create more favorable conditions for growth and development of ciliates, which resulted in higher number of total protozoa and *Entodinium* genus and increased butyrate molar proportion in rumen. The obtained differences can probably reflect various amounts of catechins (condensed tannins) present in plant materials. However, the effect of other heterogeneous compounds found in such plant additives cannot be excluded. Thus, further study on pure extract should be undertaken, but it must be considered that the price of such additives will substantially increase the cost of feed.

Nevertheless, the possibility of using native herbaceous resources in ruminants’ diet, is a step forward for the development of sustainable agriculture and is becoming more widespread in recent years.

## Figures and Tables

**Table 1:** Experiment design

Period/sheep	1	2	3	4	5	6
I (37 days)	CON	CON	VVI	VVI	QUE	QUE
II (37 days)	QUE	QUE	CON	CON	VVI	VVI
III (37 days)	VVI	VVI	QUE	QUE	CON	CON

CON, control diet; VVI, diet with lingonberry leaves addition; QUE, diet with oak bark addition.

**Table 2:** Composition of control diet for sheep^1)^

Item	Control diet
Components (% of dry matter, DM)	
Meadow hay	59.7
Barley meal	28.4
Soybean meal	9.84
Vitamin-mineral premix^2)^	2.07
Chemical composition (% of DM)	
Organic matter	94.0
Crude protein^3)^	15.2
Crude fat	2.00
Starch	24.7
NDF	51.1
ADF	26.0
ADL	3.98
Crude ash	5.85
Nutritive value^4)^	
PDI (g)	95.3
UFV (per kg)	0.80
SFU (per kg)	0.60

DM, dry matter; NDF, neutral detergent fibre; ADF, acid detergent fibre; ADL, acid detergent lignin; PDI, protein digested in small intestine; UFV, feed unit for maintenance and meat production; SFU, fill unit for sheep.

1) Diet formulated according to the ruminant nutrition recommendations [11].

2) Polfamix O-K (Trouw Nutrition Polska, Grodzisk Mazowiecki, Poland) in kg: Ca 240 g, Na 60 g, P 120 g, Mg 65 g, Zn 2.5 g, Mn 3.0 g, vit. E 1.5 g, Se 0.003 g, Co 0.015 g; vit. A 300,000 IU, vit. D_3_ 30,000 IU.

3) Expressed as N×6.25

4) Calculated for all diet components according to the values read from the tables containing information about chemical composition and nutritional value of feed [11].

**Table 3:** Concentration of ciliates (×10^4^/mL) and bacterial mass (g/8 h) in sheep rumen fluid

Item	Diet^1)^	Time (h)				SEM	D^2)^	T^2)^	D×T^2)^

		0	2	4	8				
Total ciliate	CON	94.7^a^	36.4^b^ ^x^	51.2^c^ ^x^	77.5^d^ ^x^	2.83	<0.001	<0.001	<0.001
	VVI	84.1^a^	38.5^b^ ^x^	46.4^b^ ^x^	75.3^c^ ^x^				
	QUE	93.5^a^	89.4^a^ ^y^	74.0^a^ ^y^	49.7^b^ ^y^				
*Entodinium*	CON	82.8^a^	31.3^b^ ^x^	44.6^c^ ^x^	63.3^d^ ^x^	2.47	<0.001	<0.001	<0.001
	VVI	71.8^a^	32.3^b^ ^x^	40.9^b^ ^x^	63.6^c^ ^x^				
	QUE	80.2^a^	77.2^a^ ^y^	64.1^a^ ^y^	41.4^b^ ^y^				
*Diplodinium*	CON	4.95^a^ ^x^	1.70^b^ ^x^	3.20^c^ ^x^	4.47^a^ ^x^	0.160	<0.001	<0.001	<0.001
	VVI	2.83^a^ ^y^	2.22^a^ ^xy^	1.78^b^ ^y^	4.34^c^ ^x^				
	QUE	5.18^a^ ^x^	3.00^b^ ^y^	2.60^b^ ^x^	2.55^b^ ^y^				
*Ophryoscolex*	CON	0.63^ac^ ^x^	0.14^b^ ^x^	0.44^c^	1.12^a^ ^x^	0.062	<0.001	<0.001	0.003
	VVI	1.42^a^ ^y^	0.36^b^ ^y^	0.65^c^	1.02^d^ ^x^				
	QUE	1.34^a^ ^xy^	0.26^b^ ^xy^	0.70^a^	0.63^a^ ^y^				
*Isotricha*	CON	1.08^a^ ^x^	0.44^b^ ^x^	0.84^a^	1.90^c^ ^x^	0.069	0.510	<0.001	<0.001
	VVI	1.47^a^ ^y^	0.58^b^ ^x^	0.61^b^	1.85^a^ ^x^				
	QUE	1.50^a^ ^y^	1.38^a^ ^y^	0.85^ab^	0.70^b^ ^y^				
*Dasytricha*	CON	5.23^a^	2.82^b^ ^x^	2.18^b^ ^x^	6.71^a^ ^x^	0.240	<0.001	<0.001	<0.001
	VVI	6.53^a^	3.08^b^ ^x^	2.42^b^ ^x^	4.41^c^ ^y^				
	QUE	5.24^a^	7.54^b^ ^y^	5.80^ab^ ^y^	4.40^a^ ^y^				
Bacterial mass	CON	1.27^xy^				0.062	0.003	-	-
	VVI	1.51^x^							
	QUE	1.07^y^							

SEM, standard error of the mean.

1) CON, control diet; VVI, diet with lingonberry leaves addition; QUE, diet with oak bark addition.

2) D, main effect of dietary treatment; T, main effect of time; D×T, dietary treatment and time interaction effect.

Different superscripts in a row (^a–d^ p≤0.05) differ between sampling time (0, 2, 4, 8 h).

Different superscripts in a column (^x,y^ p≤0.05) differ between diet (CON, VVI, QUE).

**Table 4:** pH, total short-chain fatty acids concentration (mM/100 mL) and molar proportions of short-chain fatty acids (%) in rumen fluid of sheep

Item	Diet^1)^	Time (h)				SEM	D^2)^	T^2)^	D×T^2)^

		0	2	4	8				
pH	CON	6.73	6.07	6.11	6.34	0.026	<0.001	<0.001	0.194
	VVI	6.70	6.15	6.05	6.33				
	QUE	6.41	5.98	6.01	6.24				
Total SCFA	CON	9.06	11.9	11.6	9.61	0.161	0.002	<0.001	0.895
	VVI	9.01	11.7	11.0	9.89				
	QUE	10.1	12.9	12.1	10.2				
Acetate	CON	63.9	64.7	65.7	65.4	0.304	0.143	0.024	0.337
	VVI	67.7	64.4	65.1	67.5				
	QUE	65.2	63.1	64.1	67.1				
Propionate	CON	15.5	21.0	19.6	17.8	0.271	<0.001	<0.001	0.234
	VVI	15.4	21.1	19.3	17.1				
	QUE	14.8	17.1	16.5	15.2				
Butyrate	CON	15.2^a^ ^y^	11.3^b^ ^x^	11.9^b^ ^x^	13.8^ab^ ^xy^	0.233	<0.001	0.066	0.011
	VVI	12.3^x^	11.4^x^	12.4^x^	12.3^x^				
	QUE	15.1^y^	15.5^y^	15.4^y^	14.3^y^				
Valerate	CON	1.31	1.43	1.45	1.16	0.031	<0.001	<0.001	0.182
	VVI	1.14	1.39	1.56	1.11				
	QUE	1.33	1.82	1.79	1.22				
Isoacids^3)^	CON	4.00^a^	1.52^b^ ^x^	1.45^b^ ^x^	1.95^c^	0.089	<0.001	<0.001	<0.001
	VVI	3.50^a^	1.67b x	1.66^b^ ^x^	1.97^b^				
	QUE	3.62^a^	2.47^b^ ^y^	2.21^b^ ^y^	2.18^b^				

SEM, standard error of the mean; SCFA, short-chain fatty acids.

1) CON, control diet; VVI, diet with lingonberry addition; QUE, diet with oak bark addition.

2) D, main effect of dietary treatment; T, main effect of time; D×T, dietary treatment and time interaction effect.

3) Sum of the isobutyric and isovaleric acids.

Different superscripts in a row (^a,b^ p≤0.05) differ between sampling time (0, 2, 4, 8 h);

Different superscripts in a column (^x,y^ p≤0.05) differ between diet (CON, VVI, QUE).

**Table 5:** Gas production in sheep rumen (mM/100 mL)

Item	Diet^1)^	Time (h)				SEM	D^2)^	T^2)^	D×T^2)^

		0	2	4	8				
Methane^3)^	CON	2.45	3.11	3.13	2.87	0.056	0.003	<0.001	0.158
	VVI	2.36	2.78	2.99	2.71				
	QUE	2.61	3.82	3.36	2.71				
Carbon dioxide^3)^	CON	4.21	5.33	5.37	4.93	0.096	0.003	<0.001	0.158
	VVI	4.05	5.23	5.12	4.64				
	QUE	4.48	6.56	5.76	4.65				

SEM, standard error of the mean.

1) CON, control diet; VVI, diet with lingonberry leaves addition; QUE, diet with oak bark addition.

2) D, main effect of dietary treatment; T, main effect of time; D×T, dietary treatment and time interaction effect.

3) Gases concentrations were calculated according to Wolin [17].
